# Methyl 2-{*N*-[2-(2,4-dichloro­phen­oxy)acet­yl]-4-[(4,6-dimeth­oxy­pyrimidin-2-yl)­oxy]anilino}propano­ate

**DOI:** 10.1107/S1600536812025494

**Published:** 2012-06-13

**Authors:** Lihong Ning, Hao Peng, Hongwu He

**Affiliations:** a Key Laboratory of Pesticide and Chemical Biology, College of Chemistry, Central China Normal University, Wuhan 430079, People’s Republic of China.

## Abstract

In the title mol­ecule, C_24_H_23_Cl_2_N_3_O_7_, the central benzene ring forms dihedral angles of 65.71 (1) and 44.42 (1)° with the pyrimidine ring and the terminal benzene ring, respectively. In the crystal, molecules are linked *via* C—H⋯O hydrogen bonds.

## Related literature

For reference bond-length data, see: Allen *et al.* (1987 ▶). For the synthesis of 4-(4,6-dimeth­oxy­pyrimidin-2-yl­oxy)benzen­amine, see: Jin *et al.* (2011 ▶). For biological properties of fungicides, see: Gozzo & Garlaschelli (1985 ▶).
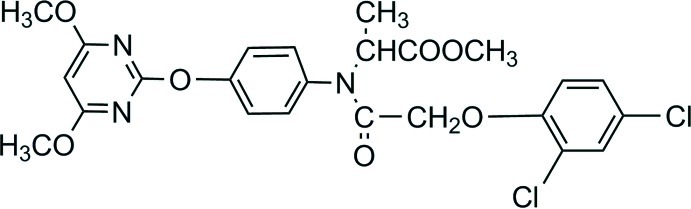


## Experimental

### 

#### Crystal data

C_24_H_23_Cl_2_N_3_O_7_
*M* *_r_* = 536.35
Triclinic, 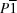

*a* = 8.2438 (9) Å
*b* = 11.2405 (12) Å
*c* = 14.2502 (15) Å
α = 85.178 (2)°
β = 78.702 (2)°
γ = 80.032 (2)°
*V* = 1273.6 (2) Å^3^
*Z* = 2
Mo *K*α radiation
μ = 0.30 mm^−1^
*T* = 298 K
0.16 × 0.12 × 0.10 mm

#### Data collection

Bruker SMART APEX CCD area-detector diffractometer
15581 measured reflections
6204 independent reflections
4390 reflections with *I* > 2σ(*I*)
*R*_int_ = 0.050

#### Refinement

*R*[*F* ^2^ > 2σ(*F* ^2^)] = 0.056
*wR*(*F* ^2^) = 0.174
*S* = 1.06
6204 reflections
329 parameters
H-atom parameters constrained
Δρ_max_ = 0.77 e Å^−3^
Δρ_min_ = −0.35 e Å^−3^

### 

Data collection: *SMART* (Bruker, 2001 ▶); cell refinement: *SAINT-Plus* (Bruker, 2001 ▶); data reduction: *SAINT-Plus*; program(s) used to solve structure: *SHELXS97* (Sheldrick, 2008 ▶); program(s) used to refine structure: *SHELXL97* (Sheldrick, 2008 ▶); molecular graphics: *PLATON* (Spek, 2009 ▶); software used to prepare material for publication: *PLATON*.

## Supplementary Material

Crystal structure: contains datablock(s) global, I. DOI: 10.1107/S1600536812025494/wn2477sup1.cif

Structure factors: contains datablock(s) I. DOI: 10.1107/S1600536812025494/wn2477Isup2.hkl

Supplementary material file. DOI: 10.1107/S1600536812025494/wn2477Isup3.cml

Additional supplementary materials:  crystallographic information; 3D view; checkCIF report

## Figures and Tables

**Table 1:** Hydrogen-bond geometry (Å, °)

*D*—H⋯*A*	*D*—H	H⋯*A*	*D*⋯*A*	*D*—H⋯*A*
C9—H9⋯O4^i^	0.93	2.57	3.402 (3)	150

Symmetry code: (i) 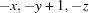.

**Fig. 1.**
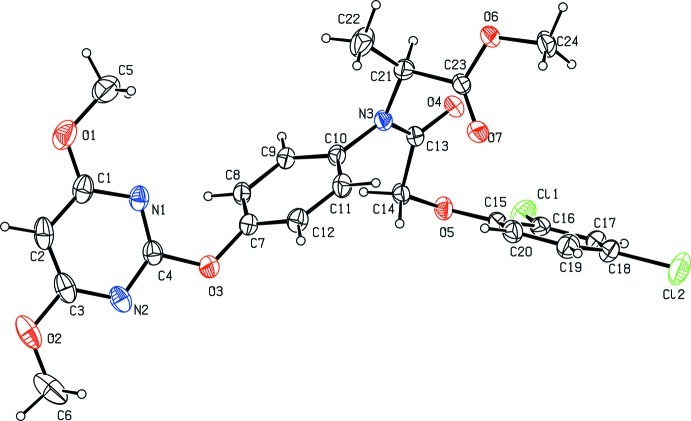
Molecular structure of the title compound, with 50% probability displacement ellipsoids. H atoms are shown as spheres of arbitrary radius.

**Fig. 2.**
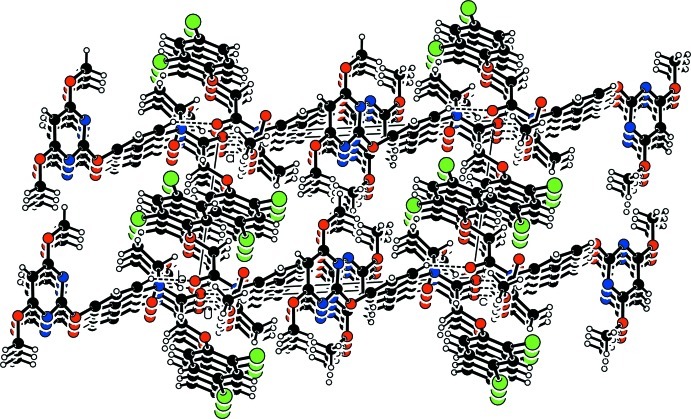
Part of the crystal packing, showing the intermolecular hydrogen bonds as dashed lines.

## supplementary crystallographic information

### Comment

*N*-acylalanine fungicides are mainly used in crop protection because of their systemic properties, with both curative and protective activity against fungal pathogens of the Peronosporales (Gozzo & Garlaschelli, 1985). The pyrimidinyl group is widely used in fungicides and drug molecular design (Jin *et al.*, 2011), so we have introduced the pyrimidinyl group into acylalanine derivatives in order to decrease resistance and increase activity.

Here we report the crystal structure of the title compound (Fig.1). The bond lengths (Allen *et al.*, 1987) and angles show normal values. The central benzene ring forms dihedral angles of 65.71 (1)° and 44.42 (1)° with the pyrimidinyl ring and the terminal benzene ring, respectively. The C9—H9···O4 intermolecular hydrogen bond plays an important role in determining the crystal structure.

### Experimental

4-(4,6-Dimethoxypyrimidin-2-yloxy)benzenamine (Jin *et al.*, 2011) (1 mmol) and methyl 2-chloropropanoate (1.2 mmol) were dissolved in 15 ml dimethylformamide, then 1 mmol K_2_CO_3_ was added with constant stirring. The temperature was maintained at 100 °C for 10 h. The reaction mixture was poured into water and extracted with ethyl acetate, dried with Na_2_SO_4_, then purified by column chromatography on silica gel with petroleum ether/ethyl acetate (4:1) to give the compound methyl 2-(*N*-(4-(4,6-dimethoxypyrimidin-2-yloxy)phenyl)amino)propanoate.

To a mixture of methyl 2-(*N*-(4-(4,6-dimethoxypyrimidin-2-yloxy) phenyl)amino)propanoate (2 mmol) and triethylamine (2 mmol) in CH_2_Cl_2_ (20 ml), 2,4-dichlorophenoxyacetyl chloride (2 mmol) was added at 2–5 °C and the mixture was stirred for another 3 h, then washed with saturated sodium hydrogen carbonate solution and dried with Na_2_SO_4_. The residue was purified by column chromatography on silica gel with petroleum ether/ethyl acetate (3:1) to give the pure title compound as a white solid. Recrystallization from ethanol over a period of one week gave colourless crystals of the title compound.

### Refinement

H atoms were geometrically positioned (Csp^2^—H = 0.93 Å, Cmethine—H = 0.98 Å, Cmethylene—H = 0.97 Å, Cmethyl—H = 0.96 Å) and refined as riding, with U_iso_(H) = xU_eq_(C), where x = 1.5 for methyl H and 1.2 for all other H atoms.

### Crystal data

**Table:**

C_24_H_23_Cl_2_N_3_O_7_	*Z* = 2
*M**_r_* = 536.35	*F*(000) = 556
Triclinic, *P*1	*D*_x_ = 1.399 Mg m^−^^3^
*a* = 8.2438 (9) Å	Mo *K*α radiation, λ = 0.71073 Å
*b* = 11.2405 (12) Å	Cell parameters from 5848 reflections
*c* = 14.2502 (15) Å	θ = 2.4–27.7°
α = 85.178 (2)°	µ = 0.30 mm^−^^1^
β = 78.702 (2)°	*T* = 298 K
γ = 80.032 (2)°	Block, colourless
*V* = 1273.6 (2) Å^3^	0.16 × 0.12 × 0.10 mm

### Data collection

**Table:**

Bruker SMART APEX CCD area-detector diffractometer	4390 reflections with *I* > 2σ(*I*)
Radiation source: fine-focus sealed tube	*R*_int_ = 0.050
Graphite monochromator	θ_max_ = 28.3°, θ_min_ = 1.8°
phi and ω scans	*h* = −10→10
15581 measured reflections	*k* = −14→14
6204 independent reflections	*l* = −18→18

### Refinement

**Table:**

Refinement on *F*^2^	Primary atom site location: structure-invariant direct methods
Least-squares matrix: full	Secondary atom site location: difference Fourier map
*R*[*F*^2^ > 2σ(*F*^2^)] = 0.056	Hydrogen site location: inferred from neighbouring sites
*wR*(*F*^2^) = 0.174	H-atom parameters constrained
*S* = 1.06	*w* = 1/[σ^2^(*F*_o_^2^) + (0.0812*P*)^2^ + 0.2621*P*] where *P* = (*F*_o_^2^ + 2*F*_c_^2^)/3
6204 reflections	(Δ/σ)_max_ < 0.001
329 parameters	Δρ_max_ = 0.77 e Å^−^^3^
0 restraints	Δρ_min_ = −0.35 e Å^−^^3^

### Special details

**Table:**

Geometry. All e.s.d.'s (except the e.s.d. in the dihedral angle between two l.s. planes) are estimated using the full covariance matrix. The cell e.s.d.'s are taken into account individually in the estimation of e.s.d.'s in distances, angles and torsion angles; correlations between e.s.d.'s in cell parameters are only used when they are defined by crystal symmetry. An approximate (isotropic) treatment of cell e.s.d.'s is used for estimating e.s.d.'s involving l.s. planes.
Refinement. Refinement of *F*^2^ against ALL reflections. The weighted *R*-factor *wR* and goodness of fit *S* are based on *F*^2^, conventional *R*-factors *R* are based on *F*, with *F* set to zero for negative *F*^2^. The threshold expression of *F*^2^ > σ(*F*^2^) is used only for calculating *R*-factors(gt) *etc*. and is not relevant to the choice of reflections for refinement. *R*-factors based on *F*^2^ are statistically about twice as large as those based on *F*, and *R*- factors based on ALL data will be even larger.

### Fractional atomic coordinates and isotropic or equivalent isotropic displacement parameters (Å^2^)

**Table:**

	*x*	*y*	*z*	*U*_iso_*/*U*_eq_	
C1	−0.1814 (4)	0.1820 (3)	0.55913 (16)	0.0750 (7)	
C2	−0.1019 (5)	0.0866 (3)	0.60971 (18)	0.0888 (9)	
H2	−0.1613	0.0343	0.6514	0.107*	
C3	0.0693 (5)	0.0735 (2)	0.59483 (17)	0.0809 (8)	
C4	0.0665 (3)	0.2312 (2)	0.49075 (15)	0.0619 (5)	
C5	−0.4294 (4)	0.3007 (4)	0.5202 (3)	0.1131 (12)	
H5A	−0.4154	0.3749	0.5442	0.170*	
H5B	−0.5467	0.2964	0.5285	0.170*	
H5C	−0.3805	0.2983	0.4533	0.170*	
C6	0.3282 (7)	−0.0322 (4)	0.6277 (3)	0.1322 (16)	
H6A	0.3745	−0.0543	0.5632	0.198*	
H6B	0.3690	−0.0945	0.6716	0.198*	
H6C	0.3610	0.0426	0.6385	0.198*	
C7	0.0948 (3)	0.3856 (2)	0.36520 (15)	0.0582 (5)	
C8	0.0267 (3)	0.34724 (19)	0.29442 (15)	0.0576 (5)	
H8	0.0106	0.2673	0.2953	0.069*	
C9	−0.0170 (3)	0.42863 (18)	0.22240 (14)	0.0529 (5)	
H9	−0.0651	0.4042	0.1749	0.063*	
C10	0.0107 (2)	0.54753 (17)	0.22072 (14)	0.0508 (4)	
C11	0.0755 (3)	0.5849 (2)	0.29330 (16)	0.0621 (5)	
H11	0.0911	0.6649	0.2932	0.075*	
C12	0.1170 (3)	0.5032 (2)	0.36626 (16)	0.0646 (6)	
H12	0.1599	0.5281	0.4157	0.077*	
C13	0.0662 (2)	0.62200 (17)	0.05341 (15)	0.0512 (4)	
C14	0.2208 (3)	0.52456 (19)	0.04013 (15)	0.0557 (5)	
H14A	0.2863	0.5293	0.0889	0.067*	
H14B	0.1868	0.4454	0.0472	0.067*	
C15	0.3997 (2)	0.63889 (18)	−0.06993 (14)	0.0522 (5)	
C16	0.4732 (2)	0.6621 (2)	−0.16498 (15)	0.0564 (5)	
C17	0.5604 (3)	0.7573 (2)	−0.19099 (17)	0.0650 (6)	
H17	0.6083	0.7721	−0.2547	0.078*	
C18	0.5757 (3)	0.8305 (2)	−0.1213 (2)	0.0724 (7)	
C19	0.5045 (3)	0.8096 (2)	−0.0276 (2)	0.0744 (6)	
H19	0.5156	0.8594	0.0189	0.089*	
C20	0.4161 (3)	0.7144 (2)	−0.00201 (17)	0.0644 (6)	
H20	0.3672	0.7011	0.0617	0.077*	
C21	−0.1595 (3)	0.7360 (2)	0.1557 (2)	0.0687 (6)	
H21	−0.2150	0.7427	0.1001	0.082*	
C22	−0.2918 (4)	0.7281 (3)	0.2402 (3)	0.1095 (12)	
H22A	−0.3292	0.6513	0.2438	0.164*	
H22B	−0.3842	0.7919	0.2355	0.164*	
H22C	−0.2486	0.7360	0.2969	0.164*	
C23	−0.0768 (3)	0.84909 (18)	0.14879 (15)	0.0550 (5)	
C24	−0.1183 (4)	1.0605 (2)	0.1175 (2)	0.0823 (8)	
H24A	−0.0790	1.0730	0.1744	0.124*	
H24B	−0.2063	1.1253	0.1070	0.124*	
H24C	−0.0275	1.0584	0.0636	0.124*	
Cl1	0.45524 (9)	0.57030 (7)	−0.25189 (5)	0.0857 (2)	
Cl2	0.68469 (11)	0.95120 (8)	−0.15360 (8)	0.1130 (3)	
N1	−0.0971 (3)	0.25673 (18)	0.49818 (12)	0.0639 (5)	
N2	0.1583 (3)	0.14535 (19)	0.53514 (13)	0.0715 (5)	
N3	−0.0284 (2)	0.62954 (15)	0.14216 (13)	0.0542 (4)	
O1	−0.3482 (3)	0.2002 (2)	0.57170 (15)	0.1022 (7)	
O2	0.1513 (4)	−0.0183 (2)	0.64236 (15)	0.1119 (8)	
O3	0.1632 (2)	0.30374 (17)	0.43166 (13)	0.0761 (5)	
O4	0.03226 (19)	0.69051 (14)	−0.01316 (11)	0.0641 (4)	
O5	0.32022 (19)	0.54002 (13)	−0.05232 (10)	0.0600 (4)	
O6	−0.1820 (2)	0.94630 (14)	0.12870 (13)	0.0702 (4)	
O7	0.0634 (2)	0.84994 (14)	0.16004 (13)	0.0695 (4)	

### Atomic displacement parameters (Å^2^)

**Table:**

	*U*^11^	*U*^22^	*U*^33^	*U*^12^	*U*^13^	*U*^23^
C1	0.0984 (19)	0.0862 (17)	0.0475 (12)	−0.0416 (15)	−0.0091 (12)	0.0017 (11)
C2	0.137 (3)	0.0818 (19)	0.0513 (13)	−0.0444 (18)	−0.0113 (15)	0.0159 (12)
C3	0.131 (3)	0.0653 (15)	0.0478 (12)	−0.0171 (16)	−0.0222 (14)	0.0056 (11)
C4	0.0814 (16)	0.0601 (13)	0.0479 (11)	−0.0198 (11)	−0.0157 (10)	0.0018 (9)
C5	0.084 (2)	0.147 (3)	0.109 (3)	−0.036 (2)	−0.0128 (18)	0.016 (2)
C6	0.172 (4)	0.110 (3)	0.093 (2)	0.044 (3)	−0.037 (3)	0.011 (2)
C7	0.0544 (11)	0.0615 (13)	0.0562 (11)	−0.0144 (9)	−0.0044 (9)	0.0091 (9)
C8	0.0619 (12)	0.0471 (11)	0.0625 (12)	−0.0166 (9)	−0.0049 (9)	0.0055 (9)
C9	0.0548 (11)	0.0507 (11)	0.0524 (10)	−0.0144 (9)	−0.0041 (8)	−0.0001 (8)
C10	0.0508 (10)	0.0454 (10)	0.0512 (10)	−0.0091 (8)	0.0024 (8)	0.0015 (8)
C11	0.0707 (14)	0.0491 (11)	0.0658 (13)	−0.0169 (10)	−0.0047 (10)	−0.0026 (10)
C12	0.0714 (14)	0.0676 (14)	0.0577 (12)	−0.0214 (11)	−0.0097 (10)	−0.0045 (10)
C13	0.0521 (10)	0.0417 (10)	0.0588 (11)	−0.0099 (8)	−0.0093 (8)	0.0053 (8)
C14	0.0563 (11)	0.0481 (11)	0.0544 (11)	−0.0052 (8)	0.0018 (9)	0.0098 (8)
C15	0.0463 (10)	0.0480 (10)	0.0555 (11)	−0.0004 (8)	−0.0037 (8)	0.0094 (8)
C16	0.0437 (10)	0.0631 (13)	0.0553 (11)	−0.0009 (9)	−0.0026 (8)	0.0052 (9)
C17	0.0448 (11)	0.0729 (15)	0.0679 (13)	−0.0074 (10)	0.0026 (9)	0.0153 (11)
C18	0.0541 (13)	0.0674 (15)	0.0905 (18)	−0.0168 (11)	0.0003 (11)	0.0081 (13)
C19	0.0710 (15)	0.0701 (15)	0.0822 (16)	−0.0164 (12)	−0.0097 (12)	−0.0049 (13)
C20	0.0667 (13)	0.0620 (13)	0.0588 (12)	−0.0073 (10)	−0.0039 (10)	0.0059 (10)
C21	0.0489 (11)	0.0513 (12)	0.0969 (17)	−0.0032 (9)	0.0011 (11)	0.0020 (11)
C22	0.0783 (18)	0.0708 (18)	0.159 (3)	−0.0151 (14)	0.0354 (19)	−0.0167 (19)
C23	0.0559 (12)	0.0469 (11)	0.0537 (11)	−0.0005 (9)	−0.0002 (9)	0.0059 (8)
C24	0.0913 (18)	0.0461 (12)	0.0980 (19)	−0.0044 (12)	−0.0036 (14)	0.0163 (12)
Cl1	0.0820 (4)	0.1117 (6)	0.0616 (4)	−0.0311 (4)	0.0092 (3)	−0.0140 (3)
Cl2	0.1001 (6)	0.0934 (6)	0.1411 (8)	−0.0505 (5)	0.0159 (5)	0.0014 (5)
N1	0.0801 (13)	0.0671 (12)	0.0487 (9)	−0.0268 (10)	−0.0111 (8)	0.0028 (8)
N2	0.1018 (16)	0.0628 (12)	0.0512 (10)	−0.0095 (11)	−0.0233 (10)	0.0038 (9)
N3	0.0520 (9)	0.0426 (9)	0.0618 (10)	−0.0045 (7)	−0.0012 (7)	0.0035 (7)
O1	0.0946 (15)	0.137 (2)	0.0777 (12)	−0.0521 (14)	−0.0042 (11)	0.0191 (12)
O2	0.174 (3)	0.0830 (14)	0.0698 (12)	−0.0018 (15)	−0.0302 (14)	0.0235 (10)
O3	0.0694 (10)	0.0823 (12)	0.0788 (11)	−0.0230 (9)	−0.0233 (8)	0.0288 (9)
O4	0.0676 (9)	0.0551 (8)	0.0673 (9)	−0.0059 (7)	−0.0180 (7)	0.0156 (7)
O5	0.0649 (9)	0.0527 (8)	0.0548 (8)	−0.0097 (7)	0.0049 (6)	0.0042 (6)
O6	0.0625 (9)	0.0498 (9)	0.0895 (11)	0.0002 (7)	−0.0083 (8)	0.0141 (8)
O7	0.0671 (10)	0.0522 (9)	0.0904 (12)	−0.0080 (7)	−0.0223 (8)	0.0041 (8)

### Geometric parameters (Å, º)

**Table:**

C1—O1	1.334 (3)	C13—N3	1.350 (3)
C1—N1	1.336 (3)	C13—C14	1.522 (3)
C1—C2	1.382 (4)	C14—O5	1.423 (2)
C2—C3	1.369 (4)	C14—H14A	0.9700
C2—H2	0.9300	C14—H14B	0.9700
C3—N2	1.326 (4)	C15—O5	1.367 (2)
C3—O2	1.342 (3)	C15—C20	1.380 (3)
C4—N1	1.315 (3)	C15—C16	1.394 (3)
C4—N2	1.317 (3)	C16—C17	1.376 (3)
C4—O3	1.363 (3)	C16—Cl1	1.721 (2)
C5—O1	1.433 (4)	C17—C18	1.379 (4)
C5—H5A	0.9600	C17—H17	0.9300
C5—H5B	0.9600	C18—C19	1.368 (4)
C5—H5C	0.9600	C18—Cl2	1.737 (2)
C6—O2	1.415 (5)	C19—C20	1.381 (3)
C6—H6A	0.9600	C19—H19	0.9300
C6—H6B	0.9600	C20—H20	0.9300
C6—H6C	0.9600	C21—N3	1.465 (3)
C7—C12	1.368 (3)	C21—C22	1.466 (4)
C7—C8	1.377 (3)	C21—C23	1.531 (3)
C7—O3	1.395 (3)	C21—H21	0.9800
C8—C9	1.376 (3)	C22—H22A	0.9600
C8—H8	0.9300	C22—H22B	0.9600
C9—C10	1.392 (3)	C22—H22C	0.9600
C9—H9	0.9300	C23—O7	1.199 (3)
C10—C11	1.378 (3)	C23—O6	1.321 (2)
C10—N3	1.439 (3)	C24—O6	1.454 (3)
C11—C12	1.383 (3)	C24—H24A	0.9600
C11—H11	0.9300	C24—H24B	0.9600
C12—H12	0.9300	C24—H24C	0.9600
C13—O4	1.216 (2)		
			
O1—C1—N1	118.8 (2)	H14A—C14—H14B	108.1
O1—C1—C2	118.8 (2)	O5—C15—C20	125.74 (18)
N1—C1—C2	122.4 (3)	O5—C15—C16	116.07 (19)
C3—C2—C1	116.1 (2)	C20—C15—C16	118.2 (2)
C3—C2—H2	121.9	C17—C16—C15	121.3 (2)
C1—C2—H2	121.9	C17—C16—Cl1	119.07 (17)
N2—C3—O2	118.4 (3)	C15—C16—Cl1	119.64 (17)
N2—C3—C2	123.6 (2)	C16—C17—C18	119.1 (2)
O2—C3—C2	118.0 (3)	C16—C17—H17	120.5
N1—C4—N2	130.0 (2)	C18—C17—H17	120.5
N1—C4—O3	118.5 (2)	C19—C18—C17	120.7 (2)
N2—C4—O3	111.5 (2)	C19—C18—Cl2	119.9 (2)
O1—C5—H5A	109.5	C17—C18—Cl2	119.39 (19)
O1—C5—H5B	109.5	C18—C19—C20	120.0 (3)
H5A—C5—H5B	109.5	C18—C19—H19	120.0
O1—C5—H5C	109.5	C20—C19—H19	120.0
H5A—C5—H5C	109.5	C15—C20—C19	120.8 (2)
H5B—C5—H5C	109.5	C15—C20—H20	119.6
O2—C6—H6A	109.5	C19—C20—H20	119.6
O2—C6—H6B	109.5	N3—C21—C22	115.3 (2)
H6A—C6—H6B	109.5	N3—C21—C23	108.87 (17)
O2—C6—H6C	109.5	C22—C21—C23	114.0 (2)
H6A—C6—H6C	109.5	N3—C21—H21	105.9
H6B—C6—H6C	109.5	C22—C21—H21	105.9
C12—C7—C8	121.4 (2)	C23—C21—H21	105.9
C12—C7—O3	116.8 (2)	C21—C22—H22A	109.5
C8—C7—O3	121.4 (2)	C21—C22—H22B	109.5
C9—C8—C7	119.2 (2)	H22A—C22—H22B	109.5
C9—C8—H8	120.4	C21—C22—H22C	109.5
C7—C8—H8	120.4	H22A—C22—H22C	109.5
C8—C9—C10	119.9 (2)	H22B—C22—H22C	109.5
C8—C9—H9	120.0	O7—C23—O6	124.5 (2)
C10—C9—H9	120.0	O7—C23—C21	125.12 (18)
C11—C10—C9	119.99 (19)	O6—C23—C21	110.36 (19)
C11—C10—N3	121.04 (18)	O6—C24—H24A	109.5
C9—C10—N3	118.97 (18)	O6—C24—H24B	109.5
C10—C11—C12	119.8 (2)	H24A—C24—H24B	109.5
C10—C11—H11	120.1	O6—C24—H24C	109.5
C12—C11—H11	120.1	H24A—C24—H24C	109.5
C7—C12—C11	119.6 (2)	H24B—C24—H24C	109.5
C7—C12—H12	120.2	C4—N1—C1	114.2 (2)
C11—C12—H12	120.2	C4—N2—C3	113.7 (2)
O4—C13—N3	122.16 (19)	C13—N3—C10	122.15 (16)
O4—C13—C14	120.86 (18)	C13—N3—C21	115.18 (17)
N3—C13—C14	116.98 (17)	C10—N3—C21	122.20 (18)
O5—C14—C13	110.13 (16)	C1—O1—C5	118.3 (2)
O5—C14—H14A	109.6	C3—O2—C6	118.4 (3)
C13—C14—H14A	109.6	C4—O3—C7	120.35 (18)
O5—C14—H14B	109.6	C15—O5—C14	117.63 (16)
C13—C14—H14B	109.6	C23—O6—C24	116.33 (18)
			
O1—C1—C2—C3	−179.1 (2)	N2—C4—N1—C1	−1.4 (4)
N1—C1—C2—C3	0.8 (4)	O3—C4—N1—C1	−178.7 (2)
C1—C2—C3—N2	−0.8 (4)	O1—C1—N1—C4	−179.9 (2)
C1—C2—C3—O2	−179.8 (2)	C2—C1—N1—C4	0.1 (3)
C12—C7—C8—C9	1.1 (3)	N1—C4—N2—C3	1.4 (4)
O3—C7—C8—C9	−170.65 (19)	O3—C4—N2—C3	178.9 (2)
C7—C8—C9—C10	1.3 (3)	O2—C3—N2—C4	178.8 (2)
C8—C9—C10—C11	−2.7 (3)	C2—C3—N2—C4	−0.2 (4)
C8—C9—C10—N3	176.97 (18)	O4—C13—N3—C10	179.28 (19)
C9—C10—C11—C12	1.8 (3)	C14—C13—N3—C10	−1.8 (3)
N3—C10—C11—C12	−177.89 (19)	O4—C13—N3—C21	−8.4 (3)
C8—C7—C12—C11	−2.0 (3)	C14—C13—N3—C21	170.50 (19)
O3—C7—C12—C11	170.1 (2)	C11—C10—N3—C13	107.0 (2)
C10—C11—C12—C7	0.6 (3)	C9—C10—N3—C13	−72.7 (3)
O4—C13—C14—O5	7.9 (3)	C11—C10—N3—C21	−64.8 (3)
N3—C13—C14—O5	−171.06 (18)	C9—C10—N3—C21	115.5 (2)
O5—C15—C16—C17	−178.33 (17)	C22—C21—N3—C13	162.8 (3)
C20—C15—C16—C17	0.1 (3)	C23—C21—N3—C13	−67.6 (3)
O5—C15—C16—Cl1	1.7 (2)	C22—C21—N3—C10	−24.9 (3)
C20—C15—C16—Cl1	−179.85 (16)	C23—C21—N3—C10	104.8 (2)
C15—C16—C17—C18	0.4 (3)	N1—C1—O1—C5	−1.0 (4)
Cl1—C16—C17—C18	−179.63 (18)	C2—C1—O1—C5	178.9 (3)
C16—C17—C18—C19	−0.4 (4)	N2—C3—O2—C6	1.2 (4)
C16—C17—C18—Cl2	−179.73 (16)	C2—C3—O2—C6	−179.7 (3)
C17—C18—C19—C20	0.0 (4)	N1—C4—O3—C7	−14.1 (3)
Cl2—C18—C19—C20	179.26 (19)	N2—C4—O3—C7	168.1 (2)
O5—C15—C20—C19	177.7 (2)	C12—C7—O3—C4	127.6 (2)
C16—C15—C20—C19	−0.6 (3)	C8—C7—O3—C4	−60.3 (3)
C18—C19—C20—C15	0.6 (4)	C20—C15—O5—C14	11.7 (3)
N3—C21—C23—O7	−20.9 (3)	C16—C15—O5—C14	−169.97 (17)
C22—C21—C23—O7	109.4 (3)	C13—C14—O5—C15	68.7 (2)
N3—C21—C23—O6	159.17 (19)	O7—C23—O6—C24	1.6 (3)
C22—C21—C23—O6	−70.5 (3)	C21—C23—O6—C24	−178.4 (2)

### Hydrogen-bond geometry (Å, º)

**Table:**

*D*—H···*A*	*D*—H	H···*A*	*D*···*A*	*D*—H···*A*
C9—H9···O4^i^	0.93	2.57	3.402 (3)	150

Symmetry code: (i) −*x*, −*y*+1, −*z*.

## References

[bb1] Allen, F. H., Kennard, O., Watson, D. G., Brammer, L., Orpen, A. G. & Taylor, R. (1987). *J. Chem. Soc. Perkin Trans. 2*, pp. S1–19.

[bb2] Bruker (2001). *SMART* and *SAINT-Plus* Bruker AXS Inc., Madison, Wisconsin, USA.

[bb3] Gozzo, F. & Garlaschelli, L. (1985). *Pestic. Sci.* **16**, 227–286.

[bb4] Jin, C. F., Liang, Y. J., He, H. W. & Fu, L. W. (2011). *Eur. J. Med. Chem.* **46**, 429–432. 10.1016/j.ejmech.2010.11.026 21144621

[bb5] Sheldrick, G. M. (2008). *Acta Cryst.* A**64**, 112–122. 10.1107/S0108767307043930 18156677

[bb6] Spek, A. L. (2009). *Acta Cryst.* D**65**, 148–155. 10.1107/S090744490804362X PMC2631630 19171970

